# Use of Translational Science, Continuous Glucose Monitoring in the Primary Care Setting for Management of Nesidioblastosis: A Case Report and Literature Review

**DOI:** 10.7759/cureus.59388

**Published:** 2024-04-30

**Authors:** Karuna Manandhar, Othman Farahneh, Ahmad Damlakhy, Ali Lattouf, Gerardo Munoz Monaco

**Affiliations:** 1 Family Medicine, Knapp Medical Center, The University of Texas Rio Grande Valley School of Medicine, Weslaco, USA; 2 Internal Medicine, Detroit Medical Center/Sinai Grace Hospital/Wayne State University, Detroit, USA

**Keywords:** pancreatic insulinoma, glucagon-like peptide 1, roux-en y, hypoglycemia, nesidioblastosis

## Abstract

Nesidioblastosis is a term used to describe histologic changes in the pancreatic cell, which are defined by beta cell hypertrophy and the formation of ductoinsular complexes. It is a disease previously most extensively identified in neonates and is a rare cause of endogenous hypoglycemia in the adult population. However, with increasing numbers of gastric bypass surgeries for the management of obesity in recent years, there has been a growing number of populations with post-gastric bypass surgery-related nesidioblastosis. Here, we will present a case of a 60-year-old female with a history of Roux-en-Y gastric bypass (RYGB) surgery who initially presented with loss of consciousness and episodes of suspected hypoglycemia. Insulinoma was ruled out, supporting the diagnosis of adult onset RYGB-associated nesidioblastosis.This article was previously presented as a poster presentation at the 2023 Research Symposium, International Conference on Health Disparities, on September 8, 2023.

## Introduction

Morbidity and mortality related to obesity are well known to the medical profession. Treatment of obesity with bariatric surgery is now recommended in patients with severe obesity, defined as a BMI ≥ 40 kg/m^2^ or ≥35 kg/m^2^ with comorbidity, as this has been shown to effectively reduce obesity-related comorbidities, hence increasing the popularity of this treatment [1]. However, one rare late complication of such treatment is severe hyperinsulinemic hypoglycemia caused by nesidioblastosis [2]. Nesidioblastosis is a term used to describe histologic changes in the pancreatic cell, which are defined by beta cell hypertrophy and the formation of ductoinsular complexes. It is a rare cause of endogenous hypoglycemia in the adult population, and definitive diagnosis requires histopathologic examination. Hence, it is a clinical diagnosis of exclusion associated with hyperinsulinemic hypoglycemia without the presence of insulinoma [3]. Treatment of nesidioblastosis includes dietary modification, medical management with octreotide, verapamil, diazoxide, and acarbose, or surgical management with pancreatectomy [4]. Recently, continuous glucose monitoring (CGM) is increasingly being used for timely notification of hypoglycemia and subsequent management [5].

## Case presentation

A 60-year-old obese female patient with no other significant chronic disease underwent RYGB surgery in 2005 for weight loss. Her medical history included gastritis and a small bowel obstruction secondary to ventral hernia, requiring surgical intervention in March 2021. She was an ex-smoker; she stopped tobacco use in November 2021, consumed alcohol socially, and has no history of illicit drug use. The patient did not take any regular medications. She had no family history of diabetes or hypoglycemia. Her BMI prior to surgery was 41 kg/m^2 ^and her BMI at the time of presentation was 27 kg/m^2^.

In March 2022, she was admitted to our hospital with an unprovoked first episode of seizure. She was referred from the Gastroenterology (GI) clinic. The patient initially mentioned that she had started to feel weak and soon suffered an episode of vomiting followed by a tonic-clonic seizure. A glucose monitor available at the time detected a blood glucose of 50 mg/dl. Dextrose 50% was administered and she was transferred to the hospital. In the emergency department (ED), the patient had a computed tomography (CT) scan of the head, which was unremarkable. The seizure was attributed to hypoglycemia, and the patient was admitted. As per the patient, she had a breakfast containing complex carbohydrates in the morning of the presentation. The patient had no previous diagnosis of diabetes both prior to and after her RYGB surgery in 2005 and had never used insulins or sulfonylureas. In the hospital, she had an electroencephalogram (EEG) which showed a normal awake routine EEG with no epileptiform or focal abnormalities; antiepileptic medications were not started at that time. The patient had a CT of the abdomen and pelvis, which showed no acute abnormalities. The patient experienced hypoglycemic episodes in the hospital and was treated with intravenous (IV) dextrose. Recommended continuous glucose monitoring (CGM). The patient was discharged on March 14, 2022, and was advised to follow up with a primary care provider.

**Figure 1:**
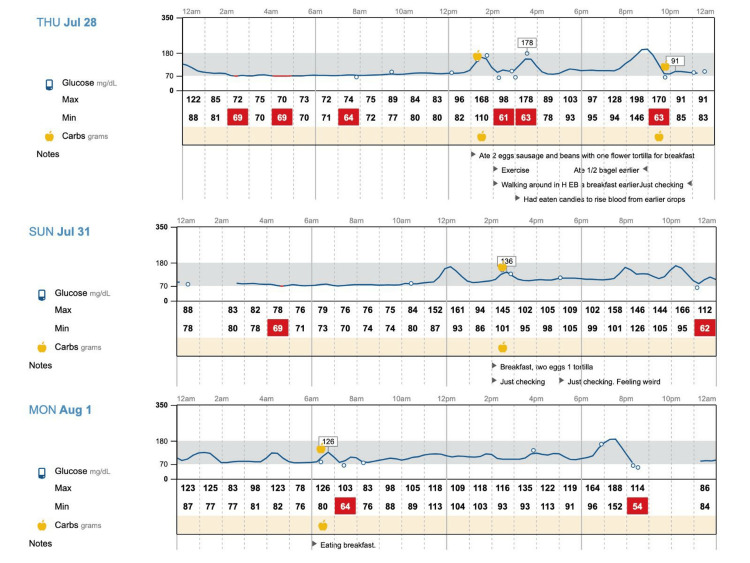
Continuous glucose monitoring A postprandial hypoglycemic episode in the patient using continuous glucose monitoring (CGM).

The patient was seen as a post-hospital discharge follow-up visit at the outpatient clinic on March 17, 2022. At the time of the presentation, the point-of-care glucose level in the clinic was 70 mg/dl. CGM was prescribed, and she started to use CGM on March 19, 2022. As a result of using CGM, the patient was able to detect multiple episodes of hypoglycemia and associated foods as possible causes of these hypoglycemic episodes. The patient described these episodes as postprandial, where after certain foods that contained complex carbohydrates such as bread, tortilla, quesadilla, and beans taco, her blood glucose level would increase instantly and then soon drop into the hypoglycemic range (see Figure 1).

**Figure 2:**
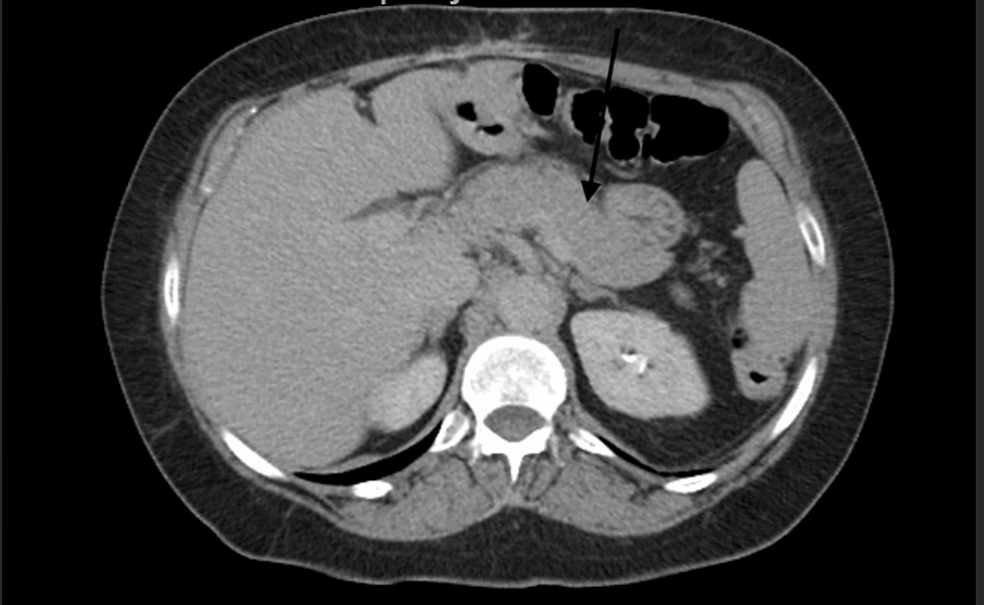
CT abdomen The black arrow demonstrates a normal pancreas.

To explore alternative causes of endogenous hypoglycemia, during her follow-up visits, simultaneous serum glucose concentration was 90 mg/dl (normal range 82-115 mg/dl) fasting Insulin and c-peptide levels were obtained, which were 4.1 uu/mL (normal range: 2.6-24.9 uu/mL) and 0.98 ng/mL (normal range: 0.80-3.85 ng/mL) respectively. Also, as part of the workup of the hospitalization in March 2022, the patient had an abdominal CT scan with oral and IV contrast, which showed a normal pancreas and ruled out any intraabdominal masses or Insulinoma (see Figure 2).

The patient also had an upper GI study for a work-up of gastritis (in November 2021, four months prior to the initial presentation) that demonstrated normal peristalsis and distensibility, excluding the diagnosis of dumping syndrome. After ruling out Insulinoma, dumping syndrome, and iatrogenic hypoglycemia, and with clinical presentation of post-prandial hypoglycemic episodes associated with complex carbohydrates, a clinical diagnosis of nesidioblastosis was considered.

Presuming the condition as nesidioblastosis, a shared decision was made for conservative management with CGM and dietary control. The patient was recommended to avoid complicated carbohydrates from her diet and have regular follow-ups. From March 2022 until December 2022, the patient experienced a total of 34 episodes of hypoglycemia and during these hypoglycemic episodes, her blood glucose levels ranged from 51-69 mg/dl. In September 2022, the patient was asked to document her meals, especially when consuming carbohydrates or when she has hypoglycemic episodes. With the help of CGM, it was noted that the majority of hypoglycemic episodes were postprandial; food that initiated these hypoglycemic episodes for our patient included bread, tortilla, quesadilla, beans taco, honey, mozzarella sticks, rice, mashed potatoes, and beer. A few hypoglycemic episodes were also noted to occur earlier in the morning (4 am-6 am), where the patient had dinner containing complex carbohydrates the night before, with CGM showing glucose level between 67-69 mg/dl in the morning. On the days when the patient did not have any carbohydrate-containing meals, it was noted that she did not have any episodes of hypoglycemia. And as the patient only started documenting her meal intake from September 2022 onwards. Regardless, we believe there is sufficient data from September to December 2022 that clearly suggests a positive link between carbohydrate intake, especially complex carbohydrates, and hypoglycemic episodes in our patient.

The patient was advised to exclude complex carbohydrates from her diet, but her December 2022 diary still showed their consumption. She experienced 10 hypoglycemic episodes during this time, prompting discussion about adding acarbose to her treatment. Additionally, the patient lost insurance coverage in December 2022, preventing her from using CGM for hypoglycemia management.

## Discussion

Nesidioblastosis has previously been widely reported in neonates. It describes persistent hyperinsulinemic hypoglycemia caused by functionally defective pancreatic beta and ductal cells. The histopathologic hallmark of nesidioblastosis includes the neoformation of Langerhans islets, hypertrophic beta-cell nuclei, and increased ductoinsular complexes [6]. In Adults, hyperinsulinemic hypoglycemia caused by adult-onset nesidioblastosis is rare and is mostly attributable to insulinomas. 

The etiology behind adult-onset nesidioblastosis is unclear, although it appears that there might be an association with RYGB surgery. With the increasing population undergoing RYGB surgery, the reported cases of RYGB-associated nesidioblastosis also seem to be increasing in frequency [2]. In obese individuals with insulin resistance, weight loss surgery leads to improved insulin sensitivity and adaptive growth of beta cells, potentially causing hypoglycemia. Non-surgical weight loss, however, is not associated with hypoglycemia or nesidioblastosis. Another explanation involves nesidioblastosis occurring post RYGB due to long-term stimulation of beta-cell growth by altered gut hormones, notably glucagon-like peptide 1 (GLP-1), which enhances insulin secretion and sensitivity [7]. Symptoms of nesidioblastosis include recurrent long-standing hypoglycemia episodes, which can often present with neurological signs such as confusion, loss of consciousness, and seizures [6]. Due to similarities in clinical presentations between nesidioblastosis and insulinoma, it can be difficult to differentiate between them. Widely available non-invasive imaging modalities such as ultrasonography (US), CT, or magnetic resonance imaging (MRI) can be equivocal [8]. Confirmatory diagnosis of adult-onset nesidioblastosis requires histopathologic examination and clinical response to therapy. However, obtaining pathology samples may not be possible in all patients, hence, in this subgroup of patients, nesidioblastosis is a clinical diagnosis of exclusion. Unlike insulinoma, which causes fasting hypoglycemia, nesidioblastosis presents with postprandial hypoglycemia within four hours of a meal and is rarely present while fasting. In addition, laboratory markers such as insulin and c-peptide levels, as well as, imaging modalities such as CT scan or MRI, can also be used to exclude insulinoma, which is a focal disease process and therefore aid in the diagnosis of nesidioblastosis which is a diffuse disease process [4]. The differential diagnosis of endogenous hyperinsulinemia hypoglycemia includes insulinoma, nesidioblastosis, and insulin autoimmune syndrome [9]. Other causes of hyperinsulinemic hypoglycemia can be due to dumping syndrome, factitious hypoglycemia, or drug-induced [10].

Our patient had her RYGB surgery in 2005 and she initially presented with hypoglycemic episodes 17 years after her surgery in 2022. She had symptomatic postprandial hypoglycemic episodes with blood glucose (BG) around 50 mg/dl, typically after eating certain food products that included bread, tortilla, quesadilla, and beans taco, however whilst fasting, her BG remained normal in the 80s mg/dl and 90s mg/dl, which is typical of nesidioblastosis. She had a CT abdomen and pelvis with intravenous contrast during her initial presentation in the hospital in March 2022, which did not show any pancreatic abnormalities or intraabdominal mass. Factitious hypoglycemia was ruled out during her hospital stay, where she continued to have hypoglycemic episodes without any exogenous insulin administration or use of hypoglycemic agents. Dumping syndrome was ruled out clinically with a supportive upper gastrointestinal (GI) study, which was performed four months prior to the initial presentation for a workup of gastritis that demonstrated normal peristalsis and distensibility. Furthermore, our patient did not have any symptoms of early dumping, which included diaphoresis, flushing, palpitations, nausea, vomiting, diarrhea, or abdominal cramps. We obtained fasting insulin and c-peptide levels, which were on the lower end of normal. These markers with negative CT findings, postprandial low blood glucose levels, and normal fasting glucose levels were used to exclude insulinoma and support the clinical diagnosis of nesidioblastosis. Moreover, in cases where imaging results are inconclusive, the presence of a positive selective arterial calcium stimulation test (SACST) exhibiting a diffuse pattern and a doubling or tripling of the basal hepatic venous serum insulin concentration indicates nesidioblastosis. Conversely, an insulinoma is commonly characterized by a focal secretion pattern [11].

We ordered CGM for our patient during her first post-hospitalization follow-up visit on Mach 17, 2022. She started using CGM on March 19, 2022, and was consistently using this to closely monitor her blood glucose levels. Since our patient was having mild to moderate symptoms of hypoglycemia and during her hypoglycemic episodes, her blood glucose levels had remained above 50 mg/dl, we opted for conservative management with nutritional modification. With CGM, our patient was able to receive prompt notifications of any hypoglycemic episodes and she was able to modify her diet accordingly. We advised our patient to reduce free carbohydrate intake, avoid any foods that trigger hypoglycemia, and space out carbohydrate intake uniformly throughout her day. Our patient was initially very active in managing her condition. She was able to identify food that triggered hypoglycemia and was able to exclude them from her diet. The patient was able to completely avoid hypoglycemic episodes on the days she avoided complex carbohydrate-containing meals. When the patient persistently followed advised dietary recommendations, there were periods of seven months(April to October) when she did not have any episodes of hypoglycemia. However, during December 2022, she had 10 episodes of hypoglycemia that correlated positively with complex carbohydrate intake, which likely explained by dietary polysaccharides found in low glycemic foods, along with the actions of GLP-1 triggered by the rapid passage of food from the stomach into the intestine and the effects of RYGB surgery, induce insulin signaling pathways, leading to heightened insulin sensitivity, which may result in a higher likelihood of hypoglycemic episodes [12-13].

This case shows that for patients with mild to moderate symptoms who have access to CGM and are motivated to make lifestyle changes, a conservative approach alone can be considered for the management of nesidioblastosis. Medical management with acarbose, diazoxide, verapamil, or octreotide is the next step in therapy for those who fail or are unable to continue with conservative management and have persistent hypoglycemia symptoms. There are clinical case reports that have demonstrated improved symptoms with medical management alone in patients with post-RYGB hypoglycemia [14-17]. For patients with severe postprandial hypoglycemia or symptoms refractory to medical treatment, surgical management with partial or subtotal pancreatectomy is the therapy of choice; however, there have been reported cases of recurrent hypoglycemia requiring total pancreatectomy [18-19].

## Conclusions

Adult-onset nesidioblastosis should be considered in all patients with unclear causes of recurrent hypoglycemia, especially in patients with a previous history of gastric bypass surgery, as it is the second most common cause of hyperinsulinemic hypoglycemia in these patients after insulinomas. For mild to moderate hypoglycemia symptoms, conservative management with strict glucose monitoring using CGM and dietary modification can be used to control hypoglycemia symptoms. However, medical or surgical management may be necessary for unsuccessful or refractory cases.
